# A Mixed-Method Assessment of a 10-Day Mobile Mindfulness Intervention

**DOI:** 10.3389/fpsyg.2021.722995

**Published:** 2021-08-31

**Authors:** Ilana Haliwa, Cameron G. Ford, Jenna M. Wilson, Natalie J. Shook

**Affiliations:** ^1^Department of Psychology, West Virginia University, Morgantown, WV, United States; ^2^NOVA Behavioral Healthcare Corporation, Goldsboro, NC, United States; ^3^School of Nursing, University of Connecticut, Storrs, CT, United States

**Keywords:** mindfulness, mobile application, app-based intervention, headspace, mood

## Abstract

Mobile mindfulness interventions represent a promising alternative to traditional in-person interventions that are resource demanding and have limited accessibility, preventing use by many populations. Despite greater accessibility and popularity of mobile mindfulness applications (apps), research is needed testing the effectiveness of brief interventions delivered *via* these platforms. The present study assessed the efficacy of a brief mobile mindfulness intervention compared to an active control for increasing state and trait mindfulness and improving mood, as well as the acceptability of the app, in a sample of undergraduate students. Participants (*N*=139; *M*_age_=19.43years, 80.6% female, 83.5% White) were randomly assigned to either a 10-day mobile mindfulness (Headspace) or cognitive training (Peak) condition. Trait mindfulness was measured pre- and post-intervention. During the 10-day intervention, participants completed 10-min daily exercises on the assigned app, responded to daily questionnaires of state mindfulness and mood, and completed a daily written log of their reactions to the app exercises. Attrition was low (90% completion rate) and did not differ by condition. Participants in the mindfulness condition spent an average of 88.15min (*SD*=24.75) meditating out of the full 100min prescribed by the intervention. State mindfulness significantly increased across the 10-day intervention for participants in the mindfulness, but not the cognitive training, condition beginning around days 5 and 6. Some aspects of trait mindfulness increased and mood improved from pre- to post-intervention, but these changes were observed in both conditions (i.e., no significant differences were observed by condition). Qualitative analysis of open-ended reactions to the mindfulness app indicated that participants reported more likes than dislikes. Common themes for likes were that participants experienced feelings of calm and focus following the daily mindfulness exercises. Dislikes included discomfort and anxiety associated with increased awareness of thoughts and physical sensations. These findings suggest that while a brief mobile mindfulness intervention is acceptable to undergraduate college students and effective at increasing state mindfulness, a longer intervention may be needed in order to elicit corresponding changes in trait-level mindfulness or mood.

## Introduction

A growing body of research demonstrates the efficacy of mindfulness interventions for improving psychological health (Baer et al., 2006; Creswell, 2017). For example, mindfulness interventions increase positive mood and decrease negative mood, improving psychological functioning (Garland et al., 2015; Gotink et al., 2016). However, traditional mindfulness interventions are time and resource intensive and thus can only be implemented in limited numbers and locations. As such, many populations are unable to receive the benefits of these interventions. A promising alternative may be mindfulness interventions administered *via* smartphone applications (apps), which do not require resources, such as access to transportation for in-person classes. Further, a commonly reported barrier to completion of traditional mindfulness interventions has been intervention length, which is typically around 8weeks (Minor et al., 2006; Carmody and Baer, 2009; Parra et al., 2019). Although abbreviated interventions are accessible *via* mobile apps, there are very few assessments of the effectiveness or efficacy and acceptability of *brief* app-based mindfulness interventions. The goal of this study was to assess the efficacy of a 10-day app-based mindfulness intervention in increasing mindfulness and improving mood, as well as qualitatively assess the acceptability of the intervention.

### Traditional Mindfulness Interventions

Mindfulness is a purposeful, non-judgmental attention to the present moment (Kabat-Zinn et al., 1985). Generally, mindfulness-based interventions involve engaging in activities or exercises that induce a state of mindfulness, through non-judgmental, present-focused attention to the physical body, emotions, and thoughts. Three common exercises are body scan, yoga, and sitting meditation. During a body scan exercise, participants are instructed to lie down with their eyes closed and sequentially direct their attention to particular areas of the body (e.g., feet, calves, abdomen, and chest; Baer et al., 2006; Cullen, 2011; Creswell, 2017). Throughout the exercise, participants are guided to notice sensations in each area, while refraining from assigning affective labels to these sensations (e.g., “my arm hurts, and that is bad”). Some forms of yoga exercise also can be used to practice body-focused mindfulness, in which participants focus on physical sensations that arise during guided gentle movement or stretching (Cullen, 2011). Finally, sitting meditation practices entail participants being guided to focus their attention on a specific stimulus, such as their breath, thoughts, or affective experience, while in a sitting position (Baer et al., 2006; Moore, 2008; Cullen, 2011; Creswell, 2017). Throughout the duration of these exercises, participants are encouraged to observe internal and external sensations non-judgmentally and to simply redirect their attention back to the focal stimulus when they find themselves distracted or engaging in judgment (Baer et al., 2006; Moore, 2008; Cullen, 2011; Creswell, 2017).

Over time, the repeated cultivation of *state* mindfulness through these exercises increases *trait* levels of mindfulness, or one’s characteristic tendency to be mindful (Moore, 2008; Kiken et al., 2015). These increases in trait mindfulness are associated with a host of salutary effects on wellbeing (Brown and Ryan, 2003; Baer et al., 2006; Garland et al., 2015). For example, greater trait mindfulness is associated with greater positive mood and lower negative mood (Brown and Ryan, 2003; Weinstein et al., 2009; Keng and Tong, 2016; Beshai et al., 2018). Furthermore, increases in trait mindfulness following mindfulness interventions have been shown to predict improvements in mood (e.g., increases in positive mood and decreases in negative mood; Brown et al., 2007; Jislin-Goldberg et al., 2012; Tamagawa et al., 2013), which may contribute to the positive effects of mindfulness interventions on psychological wellbeing (Keng et al., 2011; Creswell, 2017).

The most common mindfulness interventions tested in randomized controlled trials are modeled after the prototype of Mindfulness Based Stress Reduction (Kabat-Zinn, 1990), an 8-week mindfulness intervention consisting of 2–2.5h of weekly group mindfulness instruction and 45min per day of guided home practice (Baer et al., 2006; Creswell, 2017). Through in-person sessions and home practice, participants are taught a set of specific exercises aimed at inducing state-level mindfulness, with the ultimate goal of increasing an individual’s trait mindfulness. Overall, traditional mindfulness interventions have been consistently shown to increase trait mindfulness, improve mood, and confer benefits across a host of psychological domains (e.g., major depressive disorder, anxiety disorders, and substance use disorder; see Creswell, 2017, for a review).

However, there are significant limitations inherent in these traditional mindfulness interventions. First, participation is predicated upon having adequate time to dedicate to in-person sessions and at-home practice, as well as access to transportation, childcare, or other resources for in-person sessions. As such, time commitment has been cited as a common barrier to completion of mindfulness training programs (Chang et al., 2004; Morone et al., 2008; Simpson et al., 2019). Traditional mindfulness interventions are also generally led by trained mindfulness practitioners (≥26h of training; Crane et al., 2010), which limits availability of these interventions (i.e., number and location). Thus, there are several barriers to participating in traditional mindfulness interventions for many individuals, including those with low socioeconomic status, those residing in rural areas, and/or with limited access to transportation, childcare, or providers with mindfulness training.

### Mobile Mindfulness Apps

Barriers to traditional mindfulness interventions may be addressed by using mobile-based apps, which offer short (e.g., 5–15min) mindfulness training exercises (e.g., body scans and sitting meditation) guided by experienced instructors (Cavanaugh et al., 2014; Flett et al., 2019). The lower time and resource burden make mindfulness training accessible to a wider and more varied audience, including those for whom traditional mindfulness interventions are time or resource prohibitive (Mani et al., 2015). Further, as the didactic component of mindfulness training can be pre-recorded and delivered to users through their mobile device, app-based trainings are convenient and can be easily incorporated into users’ daily schedules. They also can reduce cost and time for researchers (e.g., clinician fees) and practitioners. This method of delivery is especially promising, given that on average 81% of US adults own a smartphone (Taylor and Silvier, 2019), and mindfulness apps are fairly popular. For example, the Headspace app (Headspace, Inc.)^1^ represents one of the most commonly used mindfulness apps with over 2 million subscribers and 65 million downloads at the time of writing (Curry, 2021).

Existing research on the efficacy of mobile mindfulness apps in increasing mindfulness and improving mood has focused largely on the Headspace app. Across 17 randomized control trials using the Headspace app, significant increases in trait mindfulness and improvements in mood (e.g., increased positive mood and decreased negative mood) have been demonstrated in samples of community adults (Howells et al., 2016; Bennike et al., 2017; Economides et al., 2018; Bostock et al., 2019; Kirk and Axelsen, 2020), university students (Noone and Hogan, 2018; Flett et al., 2019, 2020; Throuvala et al., 2020; Piil et al., 2021), employees (Nubold and Hulsheger, 2021; Piil et al., 2021; Rich et al., 2021), medical students (Wen et al., 2017; Yang et al., 2018), patients with insomnia (Low et al., 2020), cancer patients (Rosen et al., 2018; Kubo et al., 2019), and their caregivers (Kubo et al., 2019). However, there are several limitations in the present body of research.

First, most research on the use of mindfulness apps has assessed changes in trait mindfulness and mood following relatively lengthy intervention periods (e.g., ≥30days; Bennike et al., 2017; Wen et al., 2017; Noone and Hogan, 2018; Rosen et al., 2018; Yang et al., 2018; Bostock et al., 2019; Kubo et al., 2019; Flett et al., 2020; Low et al., 2020; Nubold and Hulsheger, 2021; Piil et al., 2021; Rich et al., 2021). Understanding the efficacy of shorter interventions may increase the accessibility of these interventions to a wider range of populations, especially as intervention length and time requirements are commonly reported barriers to the completion of traditional mindfulness interventions (Minor et al., 2006; Carmody and Baer, 2009; Parra et al., 2019). A few studies have tested the effects of shorter doses of mobile mindfulness interventions and have found evidence for increases in trait mindfulness (Flett et al., 2019; Kirk and Axelsen, 2020; Throuvala et al., 2020) and improvements in mood (Howells et al., 2016; Economides et al., 2018) following 10-day mindfulness interventions. Brief interventions may be an effective way to introduce mindfulness to individuals who are unfamiliar with the concept and thus may be more likely to attempt an intervention lasting 10days compared to traditional interventions lasting 30days to 8weeks. Additional research is needed to assess robustness of the efficacy of brief mindfulness interventions delivered *via* mobile apps, such as Headspace.

Further, none of the aforementioned studies have incorporated longitudinal measures of state mindfulness and mood across the intervention, but these measures are critical in order to characterize the time points at which changes in state mindfulness and mood begin to occur and to inform recommendations for intervention duration. Although trait mindfulness tends to be stable over time in the absence of intervention (Brown and Ryan, 2003; Rau and Williams, 2016), evidence suggests that trait levels of mindfulness can be augmented following repeated induction of mindful states (Garland et al., 2010; Kiken et al., 2015; Tang, 2017). Research is needed assessing day-to-day changes in state mindfulness throughout a mindfulness intervention, in order to understand the time point at which participants begin to demonstrate significant differences in state mindfulness. Similarly, while prior work has demonstrated changes in mood (i.e., increased positive mood and decreased negative mood) from pre- to post-mindfulness intervention, research is needed to map the time course of these changes across the intervention period. Understanding the time points at which significant changes in state mindfulness and mood begin to emerge will provide insight into the minimum duration required for mindfulness interventions to confer benefits for participants.

Finally, limited research exists contextualizing quantitative findings with qualitative reactions to engagement with mindfulness apps (Tomlinson et al., 2018). Specifically, while quantitative research can demonstrate the efficacy of mobile mindfulness apps in increasing mindfulness and mood, qualitative research allows researchers to better understand additional aspects of user experience, including perceived benefits and challenges associated with app use, which may contribute to these quantitative effects. Two qualitative studies have been conducted on the Headspace app (Laurie and Blandford, 2016; Mistler et al., 2017). Laurie and Blandford (2016) assessed perceptions of the Headspace app among 16 community adults instructed to use the app for 10–15min daily for 30days. Results of exit interviews were generally mixed, with participants reporting positive outcomes associated with app use (e.g., relaxation, positive mood, and improved ability to cope with negative emotions), as well as negative outcomes (e.g., uncomfortable emotions, self-judgment for not using the app more, and difficulty finding time to use the app). A second study assessed reactions to a 7-day trial of the Headspace app among a sample of 12 acute psychiatric inpatients using a mixed-methods approach (Mistler et al., 2017). Participant usability and acceptability questionnaires revealed that the majority of participants found that the app helped them focus, manage symptoms, and was easy to use. None of the participants endorsed items associated with negative outcomes or worsened symptoms. These results were supported by structured interviews in which participants reported positive outcomes associated with app use (e.g., increased ability to manage challenging emotions, better sleep, and improved mood). However, both existing qualitative studies are limited by the use of small sample sizes. In addition, both studies used semi-structured interview methodology in which participants were asked structured questions by an interviewer rather than allowing participants to reflect on their experiences using an open-ended question, in the absence of an interviewer.

Overall, initial evidence suggests that the Headspace app is generally effective at increasing trait mindfulness and improving mood. However, additional research is needed to (1) replicate effects using shorter intervention doses, (2) to characterize longitudinal changes in state mindfulness and mood across these interventions, and (3) to contextualize these findings with qualitative data on the acceptability of these interventions.

### Present Study

The purpose of the present study was to address the limitations of the existing literature on mobile mindfulness apps by assessing the efficacy of the Headspace app in increasing mindfulness and improving mood over a brief intervention period and to contextualize these results with qualitative reactions to app use. Additionally, in order to characterize the time course of changes in mindfulness and mood, we assessed state mindfulness and mood daily throughout the intervention. Trait mindfulness was assessed pre- and post-intervention, in order to determine whether a brief 10-day intervention affected trait mindfulness. Participants were randomly assigned to either a 10-day mobile mindfulness or active control condition and responded to daily questionnaires on state mindfulness, mood, and their qualitative reactions to the daily exercises. Based on prior research, we hypothesized that participants in the mindfulness condition would demonstrate greater increases in both state and trait mindfulness across the 10-day intervention, compared to the active control condition. Similarly, we expected that participants in the mindfulness condition would demonstrate greater increases in positive mood and greater decreases in negative mood across the intervention, compared to the active control condition.

## Materials and Methods

### Participants

Participants were 154 undergraduate psychology students from a university in the South Atlantic region of the United States recruited as part of a larger study examining mindfulness and self-esteem stability. Sample size for this larger study was determined using an *a priori* power analysis for a two by three mixed-model Analysis of Covariance with three groups,^2^ revealing a necessary sample size of 144 to detect a medium effect size (*f*^2^=0.20) with *α*=0.05 and power=0.80. Participants had to be 18years or older and fluent in English to participate. Because a smartphone was required for the apps, students without a mobile smartphone were ineligible to participate. Participants were randomly assigned to one of two conditions: active control (*n*=77) or experimental (*n*=77). There were seven participants in the control group and eight participants in the experimental group who did not return for the post-intervention session. Attrition did not statistically differ between control and experimental conditions (*p*=0.41), and participants who did not return post-intervention did not significantly differ from participants who completed the study on demographic variables or trait mindfulness at pre-intervention (*p*s>0.05). Thus, the final sample analyzed consisted of 139 participants (*M*_age_=19.43years, *SD*=1.26, range=18–26; 80.6% female, 74.1% White; see Table 1 for a full breakdown of demographic characteristics).

**Table 1:** Descriptive statistics and frequencies for demographic variables.

Demographic variables	*M* (*SD*)/% (frequency)
**Age (years)**	19.43 (0.05)
**Gender**	
Female	80.60% (112)
Male	18.70% (26)
Other	0.70% (1)
**Race/Ethnicity**	
White	74.10% (103)
Hispanic/Latinx	5.80% (8)
Black/African American	7.90% (11)
Asian	5.80% (8)
Native American	1.40% (2)
Other	5.00% (7)
**Year in college**	
1	41.00% (57)
2	31.70% (44)
3	20.10% (28)
4	6.50% (9)
5	0.70% (1)

### Measures

#### Mindful Attention Awareness Scale

To assess trait mindfulness pre-intervention and post-intervention, the 15-item Mindful Attention Awareness Scale (MAAS) was used. On a scale from 1 (*almost always*) to 6 (*almost never*), participants rated how frequently they had each experience (e.g., “I was finding it difficult to stay focused on what was happening”; Brown and Ryan, 2003). Items were summed to create a total score and higher scores represented higher trait mindfulness. Reliability was good for MAAS scores pre-intervention (*α*=0.78) and post-intervention (*α*=0.85).

#### Five Facet Mindfulness Questionnaire

The 39-item Five Facet Mindfulness Questionnaire (FFMQ) was used as another measure of trait mindfulness (Baer et al., 2006). It measures five facets of mindfulness: observing (e.g., “When I’m walking, I deliberately notice the sensations of my body moving.”); non-judging of inner experiences (e.g., “I criticize myself for having irrational or inappropriate emotions.”); describing (e.g., “I’m good at finding words to describe my feelings.”); acting with awareness (e.g., “I am easily distracted.”); and non-reactivity to inner experiences (e.g., “I perceive my feelings and emotions without having to react to them.”). Appropriate items were reverse scored and then items on each subscale were summed to create total subscale scores. Higher scores on each subscale indicate higher levels of trait mindfulness. Reliability was good for FFMQ scores pre-intervention (*α*=0.88) and post-intervention (*α*=0.89).

#### Toronto Mindfulness Scale

To assess state mindfulness across the 10-day intervention, the two-factor Toronto Mindfulness Scale (TMS) was used (Lau et al., 2006). The Curiosity subscale assesses wanting to learn more about one’s experiences, whereas the Decentering subscale assesses identifying thoughts and feelings and bringing these experiences to a broader awareness. On a scale from 0 (*not at all*) to 4 (*very much*), participants rated each item according to how well it described what they experienced during their daily app exercise. Six items reflected Curiosity (e.g., “I was curious to see what my mind was up to from moment to moment”), and seven items reflected Decentering (e.g., “I was aware of my thoughts and feelings without overidentifying with them”). Items were summed to compute total Curiosity (*α*’s=0.84–0.94) and Decentering (*α*’s=0.71–0.92) scores with higher scores indicating greater state mindfulness.

#### Mood Questionnaire

To assess mood across the 10-day intervention, participants rated two items twice per day (Mata et al., 2013). On a scale from 1 (*not at all*) to 7 (*very*), participants rated their positive and negative mood (i.e., “How positive are you feeling right now?” and “How negative are you feeling right now?”) each morning and each evening for 10days. The morning and evening positive ratings were averaged to create a mean positive mood score for each intervention day, and the morning and evening negative ratings were averaged to create a mean negative mood score for each intervention day. Higher scores reflected greater positive and negative mood, respectively.

#### Demographics

Participants provided basic demographics including age, gender, and race/ethnicity. Participants also reported on their experiences with mindfulness-related activities including yoga, tai chi, and meditation.

#### Mindfulness Training Intervention

Participants in the experimental, mindfulness condition engaged in a 10-day program *via* the Headspace app (see footnote 1; Santa Monica, CA, United States). Headspace is an app for smartphones designed to deliver simple daily mindfulness exercises. There is a 10-day, free program called “Basics” that allows participants to complete one daily 10-min exercise for each of the 10days.

Day 1 of the Basics program began with an animated introductory video, which reminded participants to conduct the exercises each morning at roughly the same time, sitting in a chair with an upright posture in a quiet space and informed participants that the exercises may be more difficult on some days than others. Four additional instructional videos were shown on Days 3, 5, 6, and 9, and they taught participants about mindfulness using metaphors. For example, on Day 3, participants were encouraged to view thoughts as cars on a highway. While ordinarily, people tend to chase the cars (i.e., chasing or ruminating over thoughts), with mindfulness meditation participants are instructed to try and observe the cars pass from the side of the highway. That is, the app encourages participants to view thoughts as passing events without becoming too attached to any one particular thought. Each of the animated instructional videos was between 60 and 90s long.

The Headspace mindfulness activities include exercises, such as focusing on the body, monitoring the activity of the mind, and developing non-judgmental orientation toward one’s experiences. Exercises lasted 10min each. Participants were instructed to enable Headspace to send them daily reminders to complete their exercises at a time of their choice. Participants were encouraged to complete the exercises at 9:00AM each morning. Headspace kept a log of the number of exercises participants completed, as well as the total time participants engaged in each exercise.

#### Cognitive Training Intervention

Participants in the active control condition engaged in a 10-day cognitive training intervention *via* the Peak app^3^ (London, England). Peak is an app for smartphones designed to deliver simple daily games/puzzles. There is a free program that allows participants to complete four daily games/puzzles per day. Participants can only complete each game/puzzle one time, which takes about 10min to complete. Participants engaged with unique games/puzzles each day from a random selection provided by Peak. The activities are designed to test and improve cognitive abilities including memory, attention, and processing speed. Participants were instructed to enable Peak to send them daily reminders to complete their games/puzzles. As with participants in the Headspace condition, participants were encouraged to complete the exercises at 9:00AM each morning.

The Peak app was used as an active control intervention because the structure of its free program was similar to the structure of Headspace’s program Basics. Cognitive training interventions delivered *via* smartphone apps have been used in prior research examining mindfulness-based training interventions (e.g., Bennike et al., 2017).

#### Intervention Log

Participants in both the mindfulness and cognitive training conditions were instructed to keep a paper log to record the date and time that they completed each exercise. Participants were also asked to write a brief, 1–2 sentence reaction to the exercise they engaged in that day. Participants were provided with the following instructions:

*To help keep track of your daily exercises with the app, we would like for you to complete the daily log below. Each time you complete an exercise, please record the date and time of day. Then, provide a brief reaction to the exercise. In the reaction column, please provide any information that seems relevant. This could be things that you enjoyed or didn’t enjoy about the exercise, whether the exercise was easy or difficult, or how you felt before, during, or after the exercise*.

### Procedure

Participants arrived at the laboratory and were seated at an individual workstation. Participants were told that the study’s purpose was to investigate the effectiveness of two mobile apps. Participation involved two in-lab sessions approximately 10days apart, and the completion of two daily surveys for 10days. After receiving a brief, verbal overview of the study, participants provided written consent.

During the first study session, participants completed the MAAS, FFMQ, and demographics. Next, using a random number generator in Microsoft Excel, participants were randomly assigned to one of the conditions and were instructed to download the appropriate app into their phones. They were then instructed to engage in the Day 1 exercise of their respective training. After completing the first exercise, a research assistant showed participants how to enable the daily reminder feature on their app and were instructed to complete their daily exercise at 9:00AM each morning. Participants were given a paper packet containing 10 copies of the TMS and intervention log, and they were given instructions on completing daily surveys. Participants were then thanked for their time and given course credit for their participation.

For the next 10 consecutive days, participants completed a daily, 10-min exercise on their respective app at approximately 9:00AM each morning. After completing each exercise, participants were instructed to complete the TMS and intervention log. At 10:00AM and 10:00 PM for each of the 10days, participants received a SMS message on their smartphones sent *via* SurveySignal^4^ (Hofmann and Patel, 2015) notifying them to complete the mood questionnaire. The SMS message provided them with a link to SurveyMonkey^5^ (San Mateo, California, United States).^6^ Participants were able to choose between course credit or financial compensation for completing the daily surveys. Financial compensation was awarded with $0.50 for each survey completed *via* the SurveyMonkey link, which resulted in a maximum payout of $10.00. Course credit was awarded with 0.25h of credit for each survey completed *via* the SurveyMonkey link, which resulted in a maximum five course credits.

The second study session was held in the laboratory approximately 10days after the first study session. Participants arrived at the laboratory and were seated at an individual workstation. Participants first completed the MAAS and FFMQ. Participants were instructed to return their packet with the TMS and intervention log for each of the 10days. Participants in the mindfulness intervention condition were also instructed to open the Headspace app to show the experimenter the total amount of time spent engaging in the exercises and total number of exercises completed as recorded by the app. The research assistants logged the time spent meditating for each participant. These data were not collected for the participants in the active control condition, as there was no corresponding functionality on the Peak app to record participant adherence. Participants were then debriefed and thanked for their time. Participants received course credit for their participation in the second study session.

## Results

Descriptive statistics for all study variables can be found in Tables 2 and 3. Across conditions, attrition was low (90% completion rate). Participants in the mindfulness condition spent an average of 88.15min (*SD*=24.75) meditating out of the full 100min prescribed by the intervention.^7^

**Table 2:** Descriptive statistics for trait mindfulness pre- and post-intervention.

Time point	Measures *M*(*SD*)					
	MAAS	FFMQ – observe	FFMQ – describe	FFMQ – aware	FFMQ – nonjudge	FFMQ – nonreact
Pre	3.49 (0.64)	25.60 (5.32)	24.78 (6.73)	23.67 (5.63)	23.78 (6.92)	20.15 (3.99)
Post	3.54 (0.71)	25.53 (5.47)	25.68 (6.97)	24.10 (5.62)	24.98 (6.87)	20.27 (4.04)

*MAAS, Mindful Attention and Awareness Scale; FFMQ, Five Facet Mindfulness Questionnaire*

**Table 3:** Descriptive statistics for state mindfulness and mood across study days.

Study day	Measures *M*(*SD*)			
	TMS – Curious	TMS – Decenter	Positive mood	Negative mood
1	14.39 (4.90)	13.89 (4.64)	4.63 (1.31)	2.84 (1.26)
2	13.49 (5.18)	13.70 (4.98)	4.15 (1.16)	3.67 (0.94)
3	13.30 (5.69)	13.68 (5.43)	4.82 (1.18)	2.64 (1.10)
4	13.57 (6.07)	14.23 (5.82)	4.72 (1.19)	2.70 (1.22)
5	13.56 (6.30)	14.68 (5.85)	4.76 (1.20)	2.77 (1.14)
6	14.29 (5.87)	15.72 (5.72)	4.83 (1.25)	2.75 (1.16)
7	13.96 (5.93)	15.20 (5.99)	4.91 (1.18)	2.63 (1.02)
8	14.30 (5.91)	15.76 (6.19)	4.91 (1.03)	2.62 (1.02)
9	14.55 (6.19)	15.82 (6.61)	5.10 (1.23)	2.48 (1.17)
10	15.32 (6.07)	16.61 (6.90)	5.26 (1.10)	2.34 (1.00)

*TMS, Toronto Mindfulness Scale*

### Quantitative Analyses

Missing values analysis revealed that missingness for daily responses to the mood and state mindfulness questionnaires ranged from 11.6 to 38.7%. Data were confirmed to be missing completely at random (Little’s MCAR Test *p*s>0.05). Thus, mean imputation was used to address missing data on daily assessments of mood and mindfulness for all participants.^8^

#### Changes in Trait Mindfulness

In order to assess differences in trait mindfulness between pre- and post-intervention and between experimental and active control conditions, a series of 2 (Time: pre- vs. post-intervention)×2 (Condition: experimental vs. active control) mixed-model ANOVAs were conducted. For the MAAS and the observing, awareness, and non-reactivity subscales of the FFMQ, there were no significant effects of time (*F*s<1.47, *p*s>0.23, *np*^2^<0.01), condition (*F*s<0.49, *p*s>0.49, *np*^2^<0.004), or time by condition interactions (*F*s<1.58, *p*s>0.21, *np*^2^<0.01). However, significant effects of time were revealed for both the describing [*F*(1,137)=8.02, *p*=0.005, *np*^2^=0.06] and non-judgment [*F*(1,137)=8.57, *p*=0.004, *np*^2^=0.06] subscales of the FFMQ, such that scores post-intervention (*M*=25.68, *SE*=0.59 and *M*=24.98, *SE*=0.59, respectively) was significantly higher than pre-intervention scores (*M*=24.78, *SE*=0.57 and *M*=23.78, *SE*=0.59, respectively). There were no significant effects of condition (*F*s<0.19, *p*s>0.66, *np*^2^<0.001) or time by condition interactions (*F*s<1.38, *p*s>0.24, *np*^2^<0.01) for these two subscales. In summary, while no significant differences in trait mindfulness emerged between groups pre- to post-intervention, there was evidence of increases in ability to describe and be non-judgmental from pre- to post-intervention across both groups.

#### Changes in State Mindfulness

Two 10 (Days: 1–10)×2 (Condition: experimental vs. active control) mixed-model ANOVAs were conducted to assess differences in state mindfulness between the mindfulness and active control conditions across intervention days. Condition was entered as a between-subjects variable, and day was entered as a within-subjects variable. State mindfulness as assessed by TMS curiosity and decentering subscales were entered as dependent variables.

**Figure 1:**
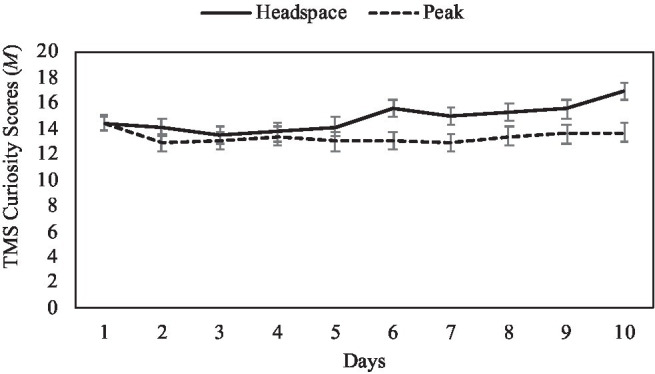
Mean TMS curiosity scores by intervention day and condition, with ±1 standard error bars.

For the TMS curiosity subscale, there was no significant main effect of condition, *F*(1,137)=3.29, *p*=0.07, *np*^2^=0.02. There was a significant main effect of day, *F*(9,129)=4.18, *p*<0.001, *np*^2^=0.23, which was qualified by a significant day by condition interaction, *F*(9, 129)=2.45, *p*=0.013, *np*^2^=0.15 (see Figure 1). Simple effects analyses revealed that TMS curiosity scores increased across the 10days for those in the mindfulness intervention condition [*F*(9,129)=5.19, *p*<0.001, *np*^2^=0.27], but not the control condition [*F*(9,129)=1.46, *p*=0.17, *np*^2^=0.09]. For participants in the mindfulness condition, significant differences began to appear in TMS curiosity scores around Day 6, such that scores on Days 6–10 were generally significantly higher than on preceding days. Specifically, for the mindfulness condition, curiosity on Days 6 and 9 was significantly higher than on Days 2–5 (*p*s<0.03); curiosity on Day 7 was significantly higher than on Days 3–4 (*p*s<0.03); curiosity on Day 8 was significantly higher than on Days 3–5 (*p*s<0.04); and curiosity on Day 10 was significantly higher than on all other days (*p*s<0.01).

**Figure 2:**
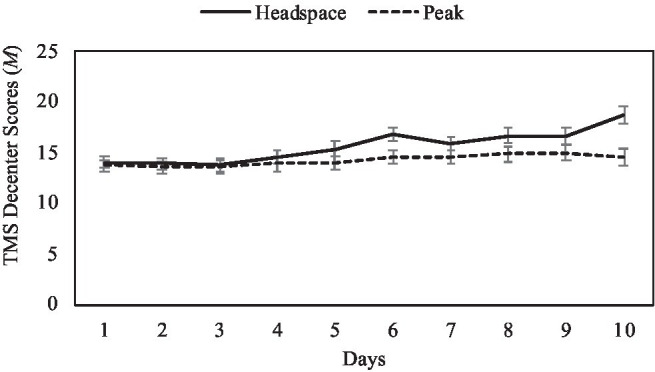
Mean TMS decenter scores by intervention day and condition, with ±1 standard error bars.

For the TMS decentering subscale, there was no significant main effect of condition, *F*(1,137)=2.98, *p*=0.09, *np*^2^=0.02. There was a significant main effect of day, *F*(9, 129)=6.11, *p*<0.001, *np*^2^=0.30. This main effect was qualified by a significant day by condition interaction, *F*(9, 129)=2.83, *p*<0.001, *np*^2^=0.17 (see Figure 2). Simple effects analyses revealed that TMS decentering scores increased across the 10days for those in the mindfulness intervention condition [*F*(9,129)=7.99, *p*<0.001, *np*^2^=0.36], but not for those in the active control condition [*F*(9,129)=0.98, *p*=0.46, *np*^2^=0.06]. For participants in the mindfulness condition, significant differences began to appear in TMS decentering scores around Day 5, such that scores on Days 5–10 were generally significantly higher than on preceding days. Specifically, for the mindfulness condition, decentering on Day 5 was significantly higher than on Days 1–3 (*p*s<0.02); decentering on Day 6 was significantly higher than on Days 1–5 and 7 (*p*s<0.04); decentering on Days 7 and 9 was significantly higher than on Days 1–4 (*p*s<0.03); decentering on Day 8 was significantly higher than on Days 1–5 (*p*s<0.04); and decentering on Day 10 was significantly higher than on all other days (*p*s<0.001).

#### Changes in Mood

Two 10 (Days: 1–10)×2 (Condition: experimental vs. active control) mixed-model ANOVAs were conducted to assess differences in mood between the mindfulness and active control conditions across intervention days. Condition was entered as a between-subjects variable, and day was entered as a within-subjects variable. Separate analyses were conducted for daily positive mood and negative mood as dependent variables.

**Figure 3:**
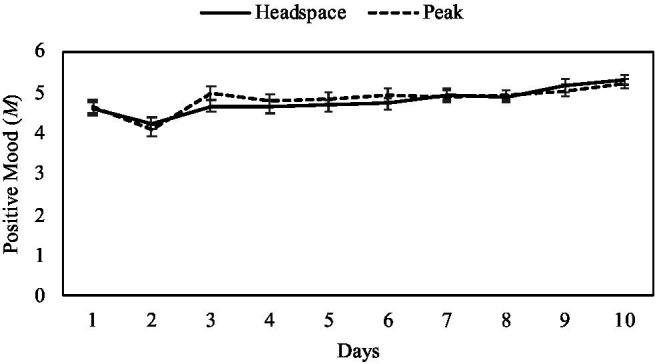
Mean positive mood scores by intervention day and condition, with ±1 standard error bars.

For positive mood, there was no significant main effect of condition [*F*(1,137)=0.129, *p*=0.72, *np*^2^=0.001]. There was a significant main effect of day [*F*(9,129)=7.46, *p*<0.001, *np*^2^=0.34], such that participants in both the mindfulness [*F*(9,129)=4.65, *p*<0.001, *np*^2^=0.325] and the control condition [*F*(9,129)=3.60, *p*<0.001, *np*^2^=0.20] demonstrated significant increases in positive mood across the 10-day intervention (see Figure 3). These significant differences began to appear on Days 9 and 10, such that positive mood on these days tended to be significantly greater than positive mood on prior days. Specifically, positive mood on Day 9 was significantly higher than on Days 1–6 and Day 8 (*p*s<0.04), and positive mood on Day 10 was significantly greater than on Days 1–8 (*p*s<0.001). Additionally, positive mood on Day 2 was significantly lower than on all other days (*p*s<0.01), and positive mood on Day 1 was significantly lower than on Days 7–10 (*p*s<0.02). There was no significant interaction of condition and day [*F*(9, 129)=0.78, *p*=0.63, *np*^2^=0.05].

**Figure 4:**
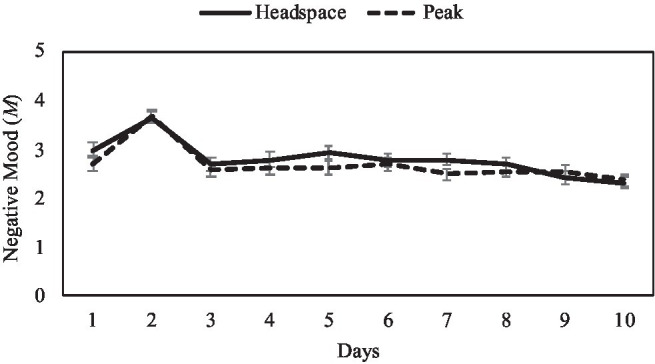
Mean negative mood scores by intervention day and condition, with ±1 standard error bars.

For negative mood, there was no significant main effect of condition [*F*(1,137)=1.06, *p*=0.31, *np*^2^=0.01]. There was a significant effect of day [*F*(9,129)=14.48, *p*<0.001, *np*^2^=0.50], such that participants in both the mindfulness [*F*(9,129)=8.36, *p*<0.001, *np*^2^=0.37] and the control condition [*F*(9,129)=7.16, *p*<0.001, *np*^2^=0.33] demonstrated significant reductions in negative mood across the 10-day intervention (see Figure 4). Across conditions, significant differences occurred most consistently at Day 10, where negative mood on Day 10 was significantly lower than on Days 1–8 (*p*s<0.001). Additionally, negative mood on Day 9 was significantly lower than on Days 5 and 6 (*p*s<0.01), and negative mood was consistently higher on Day 2 compared to all other days (*p*s<0.001). There was no significant interaction of condition and day [*F*(9, 129)=1.03, *p*=0.42, *np*^2^=0.07].

### Qualitative Analyses

Of the 77 participants in the Headspace condition, 69 participants (89.61%) returned their intervention logs with qualitative reactions to the exercises. Results from the daily mindfulness intervention log were analyzed using thematic qualitative analysis (see Braun and Clarke, 2006). First, responses were transcribed from the participant logs into an electronic document. Responses were then reviewed several times by the authors, in order to identify common themes. Following author review, the following themes were identified: likes and dislikes, ease or difficulty of use, feeling calm or relaxed, feeling focused or concentrated, and negative outcomes, broadly. Coding guidelines were developed for these themes (see Supplemental Material) and coding was performed by two independent research assistants (ICCs=0.81–0.99, *p*s<0.001). The unit of analysis for coding was independent clauses, where each clause could be coded based on multiple themes. Most daily responses consisted of 1–3 sentences.

#### Likes and Dislikes

Across all 10days of responses for the 69 participants, 56 statements from 33 participants (47.82%) were coded as “likes,” or aspects of mindfulness or using the Headspace app that they liked or enjoyed. Seventeen statements from 15 participants (21.74%) were coded as “dislikes,” or aspects of mindfulness or using the Headspace app that they disliked or did not enjoy. Given the large number of statements in this thematic category, we were also able to run quantitative analyses in the form of paired samples *t*-tests, which revealed that participants reported more “likes” (*M*=0.81, *SD*=1.07) than “dislikes” (*M*=0.25, *SD*=0.50) throughout the study, *t*(68)=4.25, *p*<0.001. Components of the mindfulness exercises that participants reported liking included the exercises themselves, positive feelings associated with meditation, and the ease of exercises. For example,

*“Today I liked the analogy about the cars…”*

*“I like the idea of not acting upon my thoughts but just letting them flow.”*

*“I liked how realistic this was compared to my previous ideas of meditation.”*

Elements of the mindfulness exercises that participants reported disliking included aspects of the exercises themselves and components of the app. For example,

*“I didn’t like being aware of my thoughts while trying to clear my mind. It didn’t help.”*

*“I didn’t like the heavy feeling in my body. It felt hard to breathe.”*

*“I didn’t really like the man’s voice ….”*

#### Ease/Difficulty of Use

Some participants specifically noted that using the Headspace app to meditate was easy (77 total mentions from 36 participants; 52.17%) or difficult (94 total mentions from 40 participants; 57.98%). Paired samples *t*-tests revealed no significant difference in the amount of references to the exercises being easy (*M*=1.12, *SD*=1.70) compared to difficult (*M*=1.36, *SD*=1.61) throughout the study, *t*(68)=−0.87, *p*=0.39. Some examples of these responses are as:

*“The exercise was very easy for me today.”*

*“The activity was very easy today.”*

*“It was difficult to clear my mind”*

*“It’s really hard for me to stay present for the whole 10-minutes – I just kept thinking about all the stuff I need to do”*

#### Calm/Relaxed

Participants also noted instances in which using the Headspace app made them feel calm or relaxed (265 total mentions from 64 participants; 92.75%). Some examples of these responses are as:

*“Very peaceful and super relaxing.”*

*“Empty, calm, free, like the ocean waves.”*

*“It was calming; almost put me to sleep.”*

#### Focused/Concentrated

Some participants reported feeling focused or concentrated as a result of using the Headspace app (48 total mentions from 23 participants; 33.33%). Some examples of these responses are as:

*“This exercise really helped me focus today and get my mind off the stress I’m dealing with.”*

*“It was helpful to watch the video that explained that the purpose of these exercises was not to completely cease the thought. I felt more focused after completing today’s exercise.”*

*“I also felt very concentrated on all the sensations my body was feeling. It had me really focus on the present moment.”*

#### Negative Outcomes

Some participants reported negative outcomes as a result of using the Headspace app, such as irritation (17 total mentions from 11 participants; 15.94%) or anxiety (5 total mentions from 4 participants; 5.80%). Some examples of these responses are as:

*“I was already very relaxed before the exercise, since it’s Saturday morning but sitting still for 10 minutes made me anxious & jittery”*

*“First needed the guidance but shortly after found “tips and tricks” annoying e.g., counting pyramid/breathing.”*

*“I didn’t enjoy today’s exercise, it aggravated me to focus on my thoughts”*

## Discussion

The present study addressed existing gaps in the literature on mobile mindfulness interventions, by assessing the efficacy and acceptability of a 10-day mobile mindfulness intervention using qualitative analyses of participant reactions to contextualize quantitative findings. State (but not trait) mindfulness increased across the 10-day intervention for participants in the experimental condition but not the control condition. For both conditions, significant increases in two facets of trait mindfulness (i.e., describing and non-judgment) and positive mood were observed from pre-intervention to post-intervention, along with decreases in negative mood. Acceptability of the intervention was generally high, with 90% of participants returning for follow-up, and relatively high rates of adherence (i.e., an average of 88.15min meditating out of the full 100min prescribed by the intervention). In qualitative reports about the mindfulness app, participants described significantly more likes than dislikes, including feelings of calmness and focus. Despite overall positive reactions, some participants reported difficulty engaging in mindfulness exercises and discomfort, anxiety, or irritability associated with present-moment attention to the body or mind.

In order to characterize the time course of changes in mindfulness, we assessed state mindfulness daily throughout the intervention. These assessments allowed us to identify the time point at which significant increases in state mindfulness began to emerge across the intervention, in order to identify the minimum intervention length required to elicit benefits. Changes in state mindfulness began to appear around Days 5 and 6 of the intervention, such that state mindfulness was significantly higher following this time point compared to the first 4days of the intervention. These findings suggest that the minimum intervention period required to observe changes in state mindfulness is as little as 5days. Notably, existing research suggests that greater state mindfulness itself is associated with a host of benefits, including greater positive emotional states and self-regulation (Brown and Ryan, 2003; Lau et al., 2006; Gayner et al., 2012). As such, even brief 5-day mindfulness interventions may prove beneficial by inducing greater states of mindfulness, along with associated benefits.

Although increases in state mindfulness were observed for the experimental condition, no significant changes in trait mindfulness were observed from pre- to post-intervention by condition. This is inconsistent with prior studies (Bennike et al., 2017; Noone and Hogan, 2018; Rosen et al., 2018; Yang et al., 2018; Flett et al., 2019, 2020; Kubo et al., 2019; Kirk and Axelsen, 2020; Throuvala et al., 2020; Nubold and Hulsheger, 2021; Piil et al., 2021; Rich et al., 2021). Differences in findings may be due to brevity of the present intervention (i.e., 10days), as the majority of previous studies ranged in length from 30days (e.g., Bennike et al., 2017; Yang et al., 2018) to 8weeks (e.g., Rosen et al., 2018; Kubo et al., 2019). However, three existing studies did find evidence for changes in trait mindfulness after only 10days of intervention (Flett et al., 2019; Kirk and Axelsen, 2020; Throuvala et al., 2020). Like our study, two of these studies utilized college student samples (Flett et al., 2019; Throuvala et al., 2020) and two used the MAAS to measure trait mindfulness (Kirk and Axelsen, 2020; Throuvala et al., 2020). Given these similarities, it is unclear what underlies the inconsistency in findings. There may be unmeasured differences in our sample as compared to the previous studies. Thus, future research is needed to replicate these findings.

Positive and negative mood were also assessed daily throughout the intervention. Positive mood significantly increased and negative mood significantly decreased across the 10-day intervention for participants in both the experimental and active control conditions, beginning largely on Days 9 and 10 of the intervention. These results may be reflective of the phenomenon known as digital placebo effects, by which benefits are derived from regular engagement with a digital device or app rather than from the intervention itself (Torous and Firth, 2016). As such, these findings may highlight the importance of utilizing active control conditions in studies assessing the effects of mobile apps. It is also possible that both app exercises were enjoyable and were effective at improving mood. Additionally, participants reported significantly greater negative mood and lower positive mood on Day 2 compared to all other study days. The cause of these significant decreases in negative mood is unclear; however, Day 2 of the intervention represents the first day that participants were asked to complete the app exercise and survey independently (Day 1 was completed in the laboratory under the guidance of a trained research assistant). Thus, it is possible that Day 2 of the intervention represents an adjustment to participants’ daily lives, requiring them to make time to complete the exercise and survey independently for the first time. Overall, effects of app use on mood were observed independent of condition, which is inconsistent with prior research (Howells et al., 2016; Economides et al., 2018). However, prior research used more comprehensive measures of mood, such as the Scale of Positive and Negative Experience (Diener et al., 2009; Economides et al., 2018) and the Positive and Negative Affect Scale (Watson et al., 1988; Howells et al., 2016). The single-item assessments of positive and negative mood may have obscured change in specific mood states or poorly tapped into current mood.

Finally, the present study also provides insight into participant reactions to engaging with a brief mobile mindfulness intervention, using a larger sample than previous studies qualitatively assessing acceptability of the Headspace app (*N*s=13–16; Laurie and Blandford, 2016; Mistler et al., 2017). As with prior studies, participants generally found the app easy to use, enjoyed the exercises, and reported feelings of calmness and focus during and following mindfulness practice (Laurie and Blandford, 2016; Mistler et al., 2017). This overall positive reaction to app use is supported by high adherence (high return rates and minutes spent meditating). However, in line with Laurie and Blandford (2016), some participants also reported difficulty engaging with the exercises and experiencing aversive emotions and sensations (i.e., anxiety, irritability, or “jitters”) during or after mindfulness practice. While these reports were less common, these findings are significant, as there are some populations for whom mindfulness practice is associated with experiences of psychological discomfort or distress, including individuals with anxiety symptoms, such as repetitive negative thinking (Schlosser et al., 2019). Indeed, within our sample, greater baseline anxiety (Beck Anxiety Inventory; Beck and Steer, 1993) significantly predicted time spent meditating in the Headspace condition (see footnote 6). As mindfulness interventions become more popular and accessible to a wider range of users, it will be critical to assess for whom the interventions may prompt aversive experiences in order to determine effective mitigation strategies.

### Limitations and Future Directions

The present study is not without limitations. First, our sample was fairly homogeneous (e.g., majority white, female, and college students), limiting generalizability. Additionally, the intervention was brief (i.e., 10-min per day for 10days), and thus, we are unable to identify the time point at which observed changes in state mindfulness might translate to corresponding changes in trait mindfulness or improved mood. Due to errors in data collection, adherence data on minutes spent meditating were only collected for 67% of participants in the mindfulness intervention group, limiting our ability to effectively assess predictors of adherence. Further, the present study utilized single-item measures of positive and negative mood, which provided a general measure of mood valence not an assessment of specific mood states. Although we chose to use single items to reduce participant burden and fatigue, we may have limited our ability to discern significant differences between groups in specific mood states. Future research is needed to replicate the present findings among a more heterogeneous sample, using more comprehensive measures of mood. This may be particularly important given that the implementation of mobile mindfulness apps has increased the accessibility of these interventions to a wider, more varied population than traditional in-person interventions. Further, longitudinally assessing state and trait mindfulness, as well as mood, across a longer intervention period may help identify the intervention length/dose necessary to observe changes in trait mindfulness. Additional research should also assess predictors of aversive experiences associated with mindfulness training in order to better tailor interventions for those populations.

## Conclusion

Mobile mindfulness interventions may represent a promising and accessible alternative to traditional in-person interventions. However, to date, limited evidence exists on efficacy and acceptability of these using brief intervention duration (i.e., <30days). The present study found that a 10-day mindfulness intervention significantly increased state, but not trait, mindfulness. These changes in state mindfulness were observed as early as Day 5 of the intervention, suggesting that even brief, 5-day mobile interventions may be sufficient to confer some benefits to participants. Although longer interventions may be necessary in order to impact trait-level mindfulness, brief interventions may be more accessible to participants with limited time and serve as an effective introduction to mindfulness practice that may translate to continued and longer-term engagement. Future research might assess changes in state and trait mindfulness across a longer intervention period, in order to identify the minimum intervention length required to observe changes in disposition and associated benefits (Brown and Ryan, 2003).

## Data Availability Statement

The raw data supporting the conclusions of this article will be made available by the authors, without undue reservation.

## Ethics Statement

The studies involving human participants were reviewed and approved by West Virginia University Institutional Review Board. The patients/participants provided their written informed consent to participate in this study.

## Author Contributions

CF and NJS contributed to conception and design of the original study. CF and IH cleaned and organized the data files and performed statistical analysis. IH wrote the first draft of the manuscript. IH, CF, JW, and NJS wrote sections of the manuscript. All authors contributed to manuscript revision, read, and approved the submitted version.

## Conflict of Interest

The authors declare that the research was conducted in the absence of any commercial or financial relationships that could be construed as a potential conflict of interest.

## Publisher’s Note

All claims expressed in this article are solely those of the authors and do not necessarily represent those of their affiliated organizations, or those of the publisher, the editors and the reviewers. Any product that may be evaluated in this article, or claim that may be made by its manufacturer, is not guaranteed or endorsed by the publisher.

## Footnotes

^1^ https://www.headspace.com

^2^The original study also included a null control group (*n* = 145). Participants in this group did not receive an intervention or complete measures of state mindfulness. Their data were used to cross-sectionally assess associations between mindfulness and self-esteem. Thus, the null control group was not included in the present study.

^3^ www.peak.net

^4^ surveysignal.com

^5^ www.surveymonkey.com

^6^In the original study, participants in the experimental, active control, and null control conditions were asked to complete the same daily online survey. Only the experimental and active control groups completed the TMS and intervention log. Thus, these were administered separately *via* printed handouts.

^7^Due to errors in data collection procedures, time spent meditating was only recorded for 67% of participants in the mindfulness training condition.

^8^The overall pattern of results of ANOVAs for changes in state mindfulness and mood did not differ when run without using mean imputation.
