# Supplementary material for: A Mixed-Method Assessment of a 10-Day Mobile Mindfulness Intervention
Source: Front Psychol. 2021 Aug 31;12:722995. doi: 10.3389/fpsyg.2021.722995 (PMC8438152; doi:10.3389/fpsyg.2021.722995)
Supplement: Supplementary file 1 [file Data_Sheet_1.docx]

Coding Guidelines

For each participant, please rate the following:

1. **Likes *about the app itself*, across all days:** For each participant, count how many times **across all days** they report liking or enjoying an aspect of the app
2. **Dislikes *about the app itself, across all days*:** For each participant, count how many times **across all days** they report disliking or not enjoying an aspect of the app

*Participant A:*

*“ I* ***liked*** *that it was very calming”*

*“ I* ***don’t like*** *scanning by body for discomfort because…”*

*“Today* ***I liked*** *the analogy about cars. I* ***didn’t like*** *how often he referred back to it”*

*“****Enjoyed*** *the calming breathing exercise”*

*“I didn’t* ***enjoy*** *the exercise today because…”*

***Likes = 3***

***Dislikes = 3***

Note, responses that include positive adjectives *not*  in reference to a specific aspect of the app should not be included:

e.g*., “Very easy, relaxing, enjoyable”*

1. **Ease/difficulty of use, by day:** For each day for each participant, please record whether they explicitly describe the exercise or use of the app as 1) easy, 2) hard, or 3) N/A (no explicit mention of ease/difficulty). Note, that if a participant expresses ease/difficulty *relative* to a prior experience (e.g., “this feels easier than yesterday), please rate as N/A. If a participant specifies ease or difficulty in relation to a specific aspect of the app, please specify this in the notes section.

*“Super easy and faster than I thought, actually relaxed me. I started yawning.” –* ***Easy***

***“****Also hard to figure our app navigation.” -* ***Hard***

***“****Medium – tired, but otherwise it went okay. “ –* ***N/A***

***“****Was hard to concentrate because of noises outside, couldn’t focus” –* ***N/A***

***“****It felt much harder this morning than yesterday. I was already very relaxed before the exercise since it’s Saturday morning but sitting still for 10minutes made me anxious & jittery” -* ***N/A***

1. **Outcomes reported, across all days**: For each participant, please count the number of times that they report the following outcomes:
2. Calming or relaxing (specific to one timepoint)

- *“I now look forward to doing this everyday. I feel much more relaxed after.”*
- *“Relaxing, calming, easy, enjoyable.”*

1. Focus (specific to one timepoint)

- *“This exercise helped me to focus on what was going on with my life. “*
- *“Helped focus on doing hw before it was due.”*

1. Negative outcomes (specific to one timepoint)

- *“Woke up to reminder, his voice made me more tired and angry to be awake. The animation was annoying to watch”*
